# Complex systems science in the AI era: a pivotal paradigm for scientific research

**DOI:** 10.1093/nsr/nwag150

**Published:** 2026-03-10

**Authors:** Mingzhe Yang, Weijie Zhao

**Affiliations:** School of Systems Science, Beijing Normal University; NSR news and science editor based in Beijing

## Abstract

Reductionism has underpinned modern science since the 17th-century Scientific Revolution. This methodology decomposes systems into minimal units and deduces wholes from parts, succeeding across physical, life and social sciences. However, reductionism reveals limits as the scientific frontier shifts from identifying building blocks to understanding how components generate collective behavior. We possess massive data and precise local equations but fail to predict cell fates, financial crises or cognitive emergence in neural networks.

Complex systems science—a mid-20th-century discipline exploring the structures, behaviors, evolution and laws of complex systems—integrates with diverse fields to transform scientific paradigms. Rapid AI development triggers this paradigm shift. AI algorithms handle massive data and complex systems, while AI systems themselves constitute complex systems whose progress depends on complex systems science.

In this *NSR* Forum, we convene five researchers to discuss the essence of complex systems science and its integration with other disciplines to support a pivotal scientific paradigm shift in the AI era.

Tingting Gao

Postdoc Researcher, Network Science Institute, Northeastern University, USA

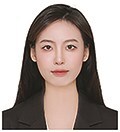

Wei Lin

Professor, School of Mathematical Sciences and Research Institute of Intelligent Complex Systems, Fudan University, China

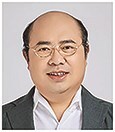

Yu Liu

Associate Professor, Department of Systems Science, Beijing Normal University at Zhuhai, China

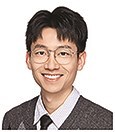

Jiang Zhang

Professor, School of Systems Science, Beijing Normal University, China

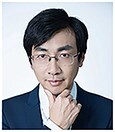

Lei Guo (Chair)

Professor, Academy of Mathematics and Systems Science, Chinese Academy of Sciences, China

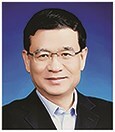

## COMPLEX SYSTEMS SCIENCE: SEEKING UNIVERSAL LAWS ACROSS SYSTEMS

**Guo:** What defines complex systems science? What role does it play in the progress of modern science and technology?

**Zhang:** As the name implies, complex systems science investigates complex systems. These systems exist everywhere. Rapid internet and AI advancements push global connectivity to new heights, transforming Earth into a unified complex system—a ‘global city’. Traditional single-discipline paradigms fail modern challenges. Complex systems science distinguishes itself by pursuing cross-domain commonalities. We extract universal concepts like ‘emergence’ and ‘evolution’ from diverse domains including economics, society, technology and healthcare. We identify universal laws such as ‘scaling laws’ and ‘power-law distributions’ and apply universal methods like ‘complex networks’ and ‘non-linear dynamics’. This approach abstracts an underlying logic across fundamental disciplines. Establishing a unified, universal methodology—specifically data-driven modeling and control, proves essential in the era of artificial intelligence (AI).

**Lin:** We are undergoing a profound scientific paradigm shift. Traditional research relies on reductionism and emphasizes ‘bottom-up’ deductive reasoning. Future science requires the integration of ‘bottom-up’ reductionism and ‘top-down’ systems theory. Iterating between these perspectives generates ‘abductive reasoning’, the core logic for future complex systems research. This view has also been articulated recently by Professor Deliang Chen.

**Guo:** In my view, complex systems science represents the frontier of systems science. The term ‘Systems Science’ uses a plural ‘Systems’ and a singular ‘Science’, implying we must extract universal scientific essences from diverse systems. This aligns with what Zhang described as ‘pursuing commonalities’.

Our research investigates universal relationships between structure, environment and function, alongside general laws governing evolution, cognition and control. Systems science emerged to address the limitations of reductionism, linear thinking and unidirectional causality within modern Western science.

**Lin:** Almost all the major scientific and engineering challenges involve complex systems. These problems feature hierarchical structures, cross-scale coupling, intense feedback and multi-agent collaboration. Therefore, complex systems research is inevitably driven by interdisciplinary topics and the integration of interdisciplinary methods. New research paradigms must emerge through deep cross-disciplinary integration to foster breakthroughs and growth of new areas.

**Liu:** Complex systems science intersects with many disciplines and continually gives rise to new research directions. For example, chaos theory reshaped weather forecasting by clarifying fundamental limits to long-term, trajectory-level precision and motivating probabilistic and ensemble-based prediction. In another case, ideas and tools from statistical physics—exemplified by the Ising model—helped inspire energy-based neural network models such as Hopfield networks and Boltzmann machines. Evolutionary computation and genetic algorithms were also widely adopted in biological modeling and optimization well before today’s deep-learning boom. In medicine, complex-systems thinking has likewise reframed key questions: cancer is increasingly studied as a tumor–host ecosystem shaped by its microenvironment, where multiscale cellular interactions are central to disease progression and therapeutic response.

In my teaching practice, I have noticed that students often engage with the most visible ‘branches’ of the field—topics such as chaos or tumor microenvironments—while overlooking their theoretical foundations. As complex systems science continues to spawn new subfields, its rapid expansion can blur its conceptual boundaries and make its core significance harder for students to grasp. This places a clear responsibility

on us as instructors. It is also a non-trivial pedagogical challenge to make the underlying throughline explicit, so that students can connect prominent applications back to foundational concepts and appreciate the coherence of the field despite its diversity.

The term ‘Systems Science’ uses a plural ‘Systems’ and a singular ‘Science’, implying we must extract universal scientific essences from diverse systems.

—Lei Guo

**Gao:** I have also observed that the distinction between complexity science and network science is not always made explicit in the literature. Network science constitutes a vital branch of complexity science, yet complexity science encompasses many other aspects such as chaos, emergence, criticality and non-linear dynamics. In practice, a substantial body of work presented under the banner of ‘network science’ is informed, sometimes implicitly, by concepts and methods from complexity science more broadly, even when this broader intellectual lineage is not foregrounded in the framing of the study.

**Zhang:** The boundary of complex systems science is indeed a problem. Many young scholars and students employ complex systems methods but misidentify them as domain-specific tools in biology or economics. In most cases, these newly developed tools are actually applicable for multi-domain approaches.

**Liu:** The cross-cutting nature of complex systems science is, in many respects, comparable to that of mathematics. While students readily recognize the value of mathematics, they often underestimate the conceptual and methodological importance of complex systems science. We can therefore borrow from mathematics education the emphasis on abstraction and structural thinking, guiding students to move from concrete, discipline-specific cases to the shared theoretical core of complex systems science.

## NEW OPPORTUNITIES IN THE AI ERA

**Guo:** AI technology is developing at a breakneck pace. What new opportunities and challenges does AI bring to complex systems research?

**Zhang:** AI and complex systems science share a deep connection. Multi-agent simulation dominated research from the 1990s through the early 2000s. Limited hardware capacity precluded large models; therefore, we defined simple rules for agents to simulate interaction and emergence in flocks or colonies.

After 2000, computer simulations based on empirical assumptions reached their limits, and we began to seek deep coupling between simulation and reality. Mobile internet and big data facilitated extensive data collection, which gave rise to network science and computational social science. Research then shifted toward mining and analyzing massive real-world datasets. This transition moved complex systems research from being rule-driven to data-driven. Currently, data-driven modeling defines the core trend.

**Gao:** Using big data and AI algorithms, now we can construct ‘virtual laboratories’ through digital twin technology. This ‘sandbox’ model reduces research costs. It facilitates long-term intervention studies and evolutionary simulations for events like disease outbreaks without causing destruction in the real world.

Challenges persist despite this promise. The mechanism of emergence remains problematic. We struggle to derive macroscopic phenomena directly from microscopic rules. Additionally, we face the challenge of coarse-graining systems to increase efficiency without sacrificing critical information or system integrity.

**Liu:** Representation learning and embedding techniques embed high-dimensional systems (language, genomes, protein sequences) into lower-dimensional semantic spaces—an opportunity that also poses new challenges for complex systems science. Tree-like phylogenies, for instance, cannot adequately represent reticulate evolution such as horizontal gene transfer. As transfer signals become increasingly visible in large-scale genomic data, complex systems science is naturally driven to develop mathematical and computational frameworks for phylogenetic networks and network-like inheritance.

Representation learning and embedding techniques embed high-dimensional systems into lower-dimensional semantic spaces—an opportunity that also poses new challenges for complex systems science.

—Yu Liu

**Guo:** Two decades ago, I identified complexity and intelligence as the core themes of systems science. Complexity research enhances our understanding of the world, while intelligence research empowers us to transform it. The current era of large models, big data, powerful computing and integrated platforms has made it possible to tackle complex problems that were once intractable.

‘AI for Science’ has emerged as a guiding paradigm. But we should notice that the scientific problems requiring deep AI intervention are, at their core, problems of complex systems. In other words, ‘AI for Complex Systems Science’ lies at the heart of ‘AI for Science’. This perspective extends naturally from natural science to engineering and social science, as complexity is a fundamental characteristic of both nature and human society.

**Lin:** From the perspective of social development, complex systems research aligns with socio-economic needs and serves as a catalyst for technological breakthroughs. While Western nations began systematic strategic planning in the 1970s, China’s research capacity—despite its strong foundation—remains fragmented across established disciplines. In the face of international competition, China urgently needs to strengthen its overall strategic layout and the organized advancement of interdisciplinary integration within complex systems science.

## INTEGRATION WITH DIVERSE DISCIPLINES

**Guo:** We noted that complex systems science merges with various disciplines to generate new theories and methods, where AI plays a vital role. Please share specific cases or insights.

**Lin:** Complex systems methods serve frontier fields like life and health. A prominent example is the research on digital virtual cells. In this area, scientists are shifting from traditional single-scale descriptions to multi-scale modeling and computation, aiming to achieve end-to-end dynamic representations of physiological states from single-cell data to tissues and even organs.

More importantly, by exploring specific problems, we can distill principled insights and formulate universal research paradigms, thereby driving the development of complexity science in general.

**Liu:** Part of my research focuses on mathematical models of the origin of life and the evolution of artificial life. Core phenomena in this area—such as oscillatory chemical dynamics and the emergence of self-replication—depend critically on network-level coupling among interacting reactions, a hallmark of complex systems. Recent work suggests that machine learning can automatically detect lifelike patterns in artificial-life simulations, potentially aiding the discovery of unfamiliar forms of living organization.

Another example comes from plasma control in a tokamak (a magnetic-confinement fusion device). Sustaining stable plasmas typically requires millisecond-scale closed-loop feedback. Under strong coupling and substantial model uncertainty, classical controllers can be difficult to tune and may generalize poorly across operating regimes. Learning-based control and hybrid approaches have begun to enable finer regulation of actuator inputs (e.g. coil voltages and auxiliary heating), improving stability and robustness during long-pulse operation.

**Gao:** I recently participated in COMPASS (Center for Complex Particle Systems), an interdisciplinary effort that bridges network science and materials science. A central motivation of the project is that network-based representations can accelerate materials simulation and development, and can further enable the design of architectures beyond traditional lattices by applying inverse methods to graph-structured information.

This network-and-interaction-centered perspective also motivates our work on aging. Historically, aging research has often focused on individual molecules or genes, yet aging cannot be fully understood by studying components in isolation; it is fundamentally a collective dynamical process that emerges from interactions among components across scales. Accordingly, we emphasize approaches that explicitly model interaction structure and system-level dynamics.

Recently, Princeton University and other institutions completed the FlyWire project, which mapped the full connectome of a fruit fly brain for the first time. This task requires 50 000 person-years manually, which is almost impossible, while AI image recognition and segmentation algorithms reduced the workload to around 33 person-years of manual proofreading.

**Zhang:** The meteorological system is a typical complex system. The chaotic nature of it makes precise weather prediction impossible with traditional analytical methods. With vast Earth system data collected by the sensor networks, now we can merge physics-informed modeling and expert knowledge with deep learning to precisely characterize the evolution process of complex weather and climate systems. This paradigm shift advances weather and climate evolution research and establishes a framework for AI-assisted complex system prediction.

Large language models (LLMs) initiate a new paradigm. We can take multi-agent simulation in social science as an example. Such simulation previously relied on simple rules and aligned poorly with reality. We now use LLMs to empower agents with human psychology and decision-making traits. This integration yields realistic simulations that can offer effective decision support. The ultimate goal of simulation is not simply ‘observe and be observed’, but to offer scientific solutions for complex real-world problems.

Humans and AI agents are integrating into a human–AI hybrid society. As agents proliferate, we should focus on the emerging concept of ‘human–AI hybrid sociology’. What impresses me a lot is that AI is evolving from a research tool into a research subject itself. The complex system formed by AI and humans represents a core research priority at this moment.

Inspired by today’s panel discussion, I just realized that we should prioritize multi-domain modeling. Current research involving AI often targets single domains like meteorology or economics. However, complexity science seeks universal laws across systems, so that multi-domain modeling may provide novel insights.

The breakthrough of LLMs relied upon the transcending of single-task limits through cross-domain learning. Similarly, deeply coupled multi-domain modeling may generate

future breakthroughs. Integrating knowledge and data from diverse fields into a larger model may facilitate a cognitive leap. Such models may identify basic commonalities across systems before humans do.

I just realized that we should prioritize multi-domain modeling … Integrating knowledge and data from diverse fields into a larger model may facilitate a cognitive leap.

—Jiang Zhang

**Guo:** The current advancement of science and technology has reached a stage where confronting systemic complexity is unavoidable. While many disciplinary fields continue to rely on a ‘case-driven’ approach to tackle specific problems, achieving deeper systemic regulation and developing a comprehensive understanding of complexity have emerged as critical challenges that demand breakthroughs. The essence of scientific progress lies precisely in the dialectical cycle of moving from the particular to the universal, and then applying the universal to guide the particular. Therefore, research on complex systems must remain deeply engaged with concrete, real-world subjects and steadfastly adhere to the principle of ‘theory rooted in practice’ to prevent the research from becoming hollow or detached.

## COMPLEX SYSTEMS RESEARCH EMPOWERING AI DEVELOPMENT

**Guo:** Let’s shift to another perspective. There is no doubt that AI itself is already regarded as a type of complex system. How does complex systems science specifically contribute to AI research?

**Zhang:** First, large AI models demonstrate the common laws of complex systems. The ‘emergence’ ability of LLMs is the best example; when the number of parameters reaches a certain threshold, its capabilities such as logical reasoning begin to emerge and surge. This transition from quantity to quality represents the phase transitions or abrupt changes common

in complex systems. Scaling law is another example. Model performance follows systematic scaling patterns as data volume or parameter size grows. This phenomenon is also a core theme in complex systems research.

Second, established methodologies of complex systems science have been introduced into AI research. Some research that impressed me a lot applied non-equilibrium statistical mechanics to model LLM training. It found that the training process mirrors the Langevin equation, with specific algorithm details corresponding to terms in the equation. This allows us to use physical theories to analyze and even optimize learning algorithms.

Furthermore, complex networks and causal analysis may play a pivotal role in tackling the interpretability challenge of AI. AI models can extract causal mechanisms from data lacking apparent causal relationships. We can employ complex networks to characterize internal patterns structurally or use causal analysis to trace and attribute model outputs to actually understand the knowledge acquired by the models.

**Lin:** First, complex systems science provides core architectures for AI. By integrating neurobiological anatomy and physiological knowledge, we can build brain-inspired computing architectures, such as spiking neural networks and integrate-and-fire dynamics. These models are driving the development of novel neural networks and AI frameworks.

Second, complex systems science provides a powerful methodology for understanding AI phenomena. Currently, critical states and critical dynamics are core research topics in complex systems, which are closely related to the mechanism of emergent intelligence in AI. Furthermore, the current development of large models relies heavily on traditional programming mindsets, focusing on achieving ‘artificial’ intelligence goals through code and resource investment. There is a relative lack of the systemic regulation mindset and the comprehensive practice of modern control methods.

**Guo:** I would also like to share a few reflections. What can complex systems science, as a discipline, offer to the development of AI? To address this question, we must first clarify the scope of the field itself. Professor Xuesen Qian, a pioneering figure in systems science in China, proposed a four-tier framework: the Philosophical Level (systems methodology), the Basic Science Level (systematology), the Technological Science Level, and the Engineering and Technology Level. In my article ‘What is Systematology’, I outlined a ‘five-theory’ structure: Systems Methodology, Systems Evolution, Systems Cognition, Systems Regulation and Systems Practice. The central three theories encompass a substantial body of quantitative research. Together, this toolbox offers a fresh perspective for understanding and designing intelligent systems.

Historically, technical practice has often preceded theory—just as the practical realization of aviation largely preceded the development of a mature, quantitative theory of aerodynamics. While AI is advancing rapidly, it currently lacks a solid theoretical foundation. As AI functions as an evolving complex system, it faces critical challenges related to safety, reliability, robustness and interpretability. Complex systems science may offer pathways toward addressing these fundamental issues.

Complex systems science provides core architectures for AI … (and) a powerful methodology for understanding AI phenomena.

—Wei Lin

## CONCERNS AND CHALLENGES IN THE AI WAVE

**Guo:** What flaws and problems have you observed in current AI and complex systems development?

**Lin:** My concern is whether this development is sustainable. Although large models and high computing power are currently mainstream, if AI is to truly permeate every aspect of human society, we must draw on the principles of complex systems—being able to both unravel intricacies and forge connections—to achieve intelligent architectures that are low-power, distributed and portable. In the long run, this more sustainable architectural design may be one of the keys to future research in intelligent science and technology.

Regarding the future direction of AI, the academic community is deeply exploring whether large models are the only path to true intelligence. I believe that the methodology of complex systems can provide strong support for the development of next-generation AI, and we are committed to applying and advancing related methods to design new intelligent frameworks and establish theories for intelligent algorithms.

**Gao:** I am also interested in efficient model execution on small, low-power devices. Sparsifying dense networks induces architectures with small-world properties and modularity. Our research demonstrates that recurrent neural networks evolve structural features resembling the human brain when training optimizes both 3D connection costs and accuracy. This fusion of complexity science and AI can generate breakthroughs. The FlyWire project can serve as an example. Insights from neuroscience, network science and developmental biology inspired efficient architectures to transcend scaling laws. Such cross-disciplinary integration is the main path for ‘Embodied AI’, and establishes foundations for next-generation adaptive systems.

**Zhang:** Energy consumption poses a technical challenge, yet AI’s deep societal integration creates a more fundamental concern. This integration triggers serious chain reactions. On the one hand, exponential energy consumption growth intensifies global power shortages and environmental stress, pushing planetary resource limits. On the other hand, technological shifts bring unemployment risks to employees in multiple areas, from basic industrial workers to white-collar workers. These environmental, resource and social issues require attention from not only the technical perspective, but also a social evolution perspective. Complex systems science should provide theoretical support for analyzing AI’s environmental and social impacts.

**Liu:** The term ‘AI slop’ refers to training language models on AI-generated content. Evidence suggests that secondary training on such synthetic data can degrade performance. Today’s LLMs owe much of their capability to high-quality human corpora accumulated over centuries, so an increasingly practical problem is to identify and filter generative text within training pipelines.

Since 2020, explosive and uncontrollable information flows have reshaped societal attention and intensified echo chamber effects.

—Tingting Gao

Long-term memory is another bottleneck. Performance often deteriorates as context grows, while naive external storage and retrieval can introduce redundancy and fragmentation. A promising direction is to treat memory not as ‘more tokens’, but as structured compression, building representations that preserve predictive and causal structure while discarding repeated or low-value detail. Importantly, this compression should not be delegated to the LLM itself in a circular way; it calls for more fundamental principles—particularly information theory and algorithmic information theory, which are among the core theoretical pillars of complex systems science—to determine what should be retained, at what granularity, and under what fidelity constraints.

**Gao:** Catastrophic forgetting warrants attention. Unlike humans, who acquire and accumulate knowledge, neural networks lose old memories when learning new skills. From a complex systems perspective, researchers often view neural networks as dynamical systems in high-dimensional parameter spaces, with different tasks representing distinct attractors. Introducing new tasks destabilizes existing attractors, causing catastrophic forgetting. To mitigate this, we can constrain gradient descent directions to prevent excessive shifts in key parameters, or introduce modular mechanisms to activate new modules while shielding interference from old tasks, ensuring new and old knowledge coexist during evolution. Complex systems science has been driving AI research. Breakthroughs like ant colony algorithms and diffusion models originated in this way.

We also face risks from algorithmic over-feeding. Since 2020, explosive and uncontrollable information flows have reshaped societal attention and intensified echo chamber effects. Complex systems theory can be applied to identify and regulate these abnormal information flows.

